# The Use of Epigenetic Biomarkers as Diagnostic and Therapeutic Options

**DOI:** 10.3390/epigenomes6040030

**Published:** 2022-09-27

**Authors:** Le Zhang, Emma M. Rath, Yuen Yee Cheng

**Affiliations:** 1 Institute for Biomedical Materials and Devices (IBMD), Faculty of Science, University of Technology Sydney, Sydney, NSW 2007, Australia; 2 Giannoulatou Laboratory, Victor Chang Cardiac Research Institute, Darlinghurst, NSW 2010, Australia

The last few decades have brought tremendous advances in the mechanisms of epigenetic regulation, with DNA methylation, histone methylation and acetylation, microRNAs and other noncoding RNAs being among the most prominent. The role of epigenetics as a breakthrough discipline in biomedicine aims to improve precision medicine, provide new epigenetic biomarkers, investigate therapeutic targets, and develop novel epigenetic drugs. The advances in this field are not only proving to be catalytic for our basic understanding of epigenetic regulation, but are also accelerating the use of epigenetic biomarkers in the development of new diagnostics and therapies that are rapidly finding their way into clinics.

This Special Issue of the *The Use of Epigenetic Biomarkers as Diagnostic and Therapeutic Options 2.0* on *Epigenomes* celebrates these developments by bringing together a number of research articles and reviews that focus on clinical opportunities for the detection and diagnosis of different diseases using epigenetic biomarkers. This collection of thought-provoking articles highlights the remarkable transformation that the field has undergone, but also touches on the many technical and conceptual challenges going forward. Topics of interest included the pilot study of RNA methylation in COVID-19 patients’ samples, epigenetic signatures of centrosomes in cancer diagnosis, epigenetic analyses of alcohol consumption, epigenetic approaches for rare diseases diagnosis and early cancer detection, and many more.

This Special Issue contains two articles that focus on the application of epigenetic biomarkers in clinical practices. This breadth highlights the diversity of the field, with researchers working on biomarker development and mechanistic understanding. This interplay is captured in one research article and two reviews that have witnessed significant progress over the last few years. Jorge Luis Batista-Roche et al. report that the level of global m6A RNA methylation differs in COVID-19 patients’ samples infected by SARS-CoV-2 variants. For example, m6A levels were significantly lower in the variants of concern (VOC) delta- and omicron-positive individuals compared to non-infected individuals and individuals with complete vaccination schemes showed significantly lower m6A levels than unvaccinated individuals [1]. One of the reviews (Beatriz Martinez-Delgado et al.) [2] discusses epigenomic approaches for the diagnosis of rare diseases that are based on functional aspects of the genome, including studies that have successfully provided diagnoses for complex undiagnosed cases. Another review by the group of Matt Trau (Nicolas Constantin et al.) collates the latest and most promising ingle test for multi-cancer early detection (stMCED) methodologies, summarizes their clinical performances, and discusses the specific requirements multi-cancer tests should meet to be successfully implemented into screening guidelines [3].

The centrosome is a non-membranous organelle comprised of centrioles surrounded by pericentriolar material, whose abnormalities have been linked to multiple solid tumors and hematological malignancies. To understand the epigenetic signatures of centrosome-related genes in cancers is a challenge, but prospective in cancer diagnosis. In this Issue, Zhou Zhang et al. explore the potential role of DNA methylation, a critical epigenetic modification, of centrosome-related genes in different cancers, which will enhance the understanding of the epigenetic mechanisms underlying the DNA methylation of centrosome-related genes in cancers [4].

Two papers in this Special Issue describe the relationship between epigenetic signatures and diseases in order to gain new insight into molecular and cellular mechanisms. Kelsey Dawes et al. explore epigenetic analyses of alcohol consumption in combustible and non-combustible nicotine product users in a biochemically verified cohort of exclusive cigarette smokers [5]. Shreya Sarkar et al. present the current scenario of cardiovascular diseases and congenital heart defects associated with COVID-19 patients and provide a developmental biology perspective of the roles of angiotensin-converting enzyme 2 (ACE2) and its axis members [6].

It has been almost a centenary since the hypothesis of epigenetic changes that affect the expression of chromosomes was put forth by Nikolai Koltsov. In the last few decades, epigenetic biomarkers have significantly contributed to our improved understanding of the origins and progression of disease. Moreover, increasing evidence shows that epigenetic biomarkers have potential for personalized medicine. With the development of these achievements, this Special Issue aims to propel us further on this exciting journey of discovery.
